# Detection of Low Abundance RNA Molecules in Individual Cells by Flow Cytometry

**DOI:** 10.1371/journal.pone.0057002

**Published:** 2013-02-18

**Authors:** Mary Beth Hanley, Woodrow Lomas, Dev Mittar, Vernon Maino, Emily Park

**Affiliations:** Research and Development, BD Biosciences, San Jose, California, United States of America; University of Crete, Greece

## Abstract

A variety of RNA analysis technologies are available for the detection of RNA transcripts from bulk cell populations. However, the techniques for RNA detection from individual cells have been limited. Here we adapt a novel *in situ* signal amplification method (the RNAScope® detection platform) for the analysis of intracellular RNAs in individual cells by flow cytometry. Using novel target-specific probes that were designed to suppress background signals, we demonstrate the specific detection of HIV gag RNAs in HIV-infected cellular samples, in addition to bcr and abl mRNAs in the K562 cell line. This method was capable of distinguishing cells expressing low abundance RNA transcripts and correlated well with quantitative imaging analysis. Furthermore, multiple distinct RNA targets were simultaneously detected with a high specificity without interference. Overall, the sensitivity and specificity of this method will be useful for the analysis of functionally important RNA species from individual cells, even at very low copy numbers.

## Introduction

Microarrays and quantitative PCR are powerful tools for gene expression analysis that have facilitated our understanding of the intricate biology of normal and disease-state cells and tissues [1]–[4]. Moreover, with the recent advances in high-throughput sequencing technologies, transcriptome profiling by RNA-seq delivers comprehensive gene expression analysis with a large dynamic range [5], [6]. The NanoString**®** Technologies’ nCounter gene expression system reports to have similar sensitivity and accuracy as real-time PCR and includes multiplexing capabilities [7]. These technologies provide the ability to understand the function of genes of interest and also to identify gene expression signatures that distinguish altered biological events from normal events. However, most gene expression studies have used bulk measurements from heterogeneous cells and tissues, in which information from rare or specific cell types can be obscured. By analyzing gene expression in individual cells, a more complete picture of the gene expression dynamics within heterogeneous samples can be captured [8]–[11].

Many single cell analysis tools have been developed and are increasingly applied to address these complex questions [12]–[17], each with its own limitations. Recently RNA-Seq and Fluidigm technologies introduced methods utilizing next generation sequencing or a PCR-based approach allowing for gene expression analysis in single cells, however, these methods require that single cells be isolated prior to analysis[18]–[20]. Flow cytometry, on the other hand, allows for simultaneous measurements of many biomarkers in individual cells in bulk populations. However, such analysis has been limited primarily to proteins and total DNA or highly abundant DNA sequences [21]. Although fluorescent *in situ* hybridization (FISH) technologies have been attempted for high-throughput intracellular RNA analysis by flow cytometry [22]–[24], only limited applications such as acute viral infection or cellular markers with abundant RNA expression have been demonstrated. Since most gene transcripts are present in low quantities (less than 50 copies per cell) [25], the specificity and sensitivity of these RNA FISH technologies are inadequate for the analysis of a broad range of specific gene expression patterns in individual cells.

Recently, a modified form of branched DNA technology has been developed which allows for the visualization of single RNA molecules in cells by image cytometry [26], [27]. Based on the same probe design approach, Wang et al recently reported a novel *in situ* hybridization technology platform (RNAScope) to analyze individual RNA molecules in formalin-fixed, paraffin-embedded tissues [27]. The unique probe design contains paired target probes that form a z-design platform for sequential hybridization-mediated signal amplifications. This strategy allows the visualization of single RNA molecules in cells by simultaneous signal amplification of multiple RNA targets. In addition, the target probe design includes a unique tail sequence type for each probe in the target probe pair that greatly decrease the likelihood of nonspecific hybridizations to occur, yielding superior background suppression.

This report describes the adaptation of RNAScope technology for cell analysis in suspension to enable RNA flow cytometry for single cell analysis of intracellular RNAs in two model systems. The specificity of RNA flow cytometry was confirmed with HIV gag RNA detection in HIV-infected cells. Sensitivity was also established so that the cells expressing specific mRNAs can be separated from the background even when the mRNAs are expressed at very low copy numbers. We also demonstrated the potential utility of multiplexing various RNA targets; using bcr and abl as cellular targets, by multiparametric RNA flow cytometry. The RNA flow cytometry assay described in this article represents a valuable tool for the specific and sensitive detection of multiple RNA transcripts from single cells in heterogeneous biological specimens.

## Methods

### Ethics Statement

An Institutional Review Board approved informed consent form was used to obtain written informed consent from each blood donor prior to the initiation of these investigational studies.

### Cell maintenance

Cell lines H9, H9IIIB, and K562 were acquired from the American Type Culture Collection (ATCC). Cells were maintained in I10 medium containing Iscove’s Modified Dulbecco’s Medium (Life Technologies) supplemented with 10% fetal bovine serum (Life Technologies), and 1% (v/v) penicillin, streptomycin, and L-glutamine (Life Technologies).

Normal whole blood was collected in EDTA BD Vacutainer® collection tubes (BD) through the BD in-house donor program (BD Biosciences, San Jose, CA). Peripheral blood mononuclear cells (PBMCs) were isolated using Histopaque®-1077 (Sigma). For the cryopreservation of PBMCs, cells were washed and resuspended in FBS supplemented with 10% DMSO (EMD Chemicals), the vials placed in a Cryo 1°C freezing container (Nalgene), and subsequently stored at -80°C for 24 hours prior to transfer to liquid nitrogen. Frozen PBMC samples were quickly thawed at 37°C and transferred to 50-ml polypropylene conical tubes, diluted drop-wise in I10 medium, and then centrifuged at 1,500 *g* for 10 minutes.

### Viral infection of PBMCs

Prior to HIV-1 infection, PBMCs were activated in I10 medium containing 1 µg/ml of phytohemagglutinin (PHA; Sigma). Cells were cultured in PHA-containing medium at 2×10^6^cells/ml for 2 to 4 days at 37°C with 5% CO_2_ to allow blasting. PHA-PBMC blasts (10×10^6^ cells/ml) were infected with HIV-1 viral stock of NL43 (Advanced Biotechnologies Inc.) at an MOI of 0.01 in I10 medium. Cells were incubated with the virus for 2 hours at 37°C and 5% CO_2_, at which point I10 medium containing 50 U/ml of interleukin-2 (Roche) was added to bring the cell concentration to 2 10^6^ cells/ml. A mock infection, in which no virus was added to the blasts, was also set up as a control. Cells were checked at regular intervals for HIVp24 antigen by flow cytometry (per the method described in the following section) to determine when the infected cells were optimal for use in experiments.

### HIVp24 flow cytometry

Cells that were intracellular p24 antigen stained were washed with X-Vivo™ 15 medium (Lonza), resuspended in 1% paraformaldehyde (Electron Microscopy Sciences), and incubated for 10 minutes at room temperature. Cells were then centrifuged and resuspended in a permeabilization buffer containing 0.2% saponin (Sigma) in 1X BD FACS™ lysing solution (BD Biosciences) and incubated for 15 minutes at room temperature. After another centrifugation, the cell pellet was resuspended in X-Vivo 15 medium, an anti-p24 PE mAb (BD Biosciences custom conjugation of 37 g12 (Polymun)) was added, and the cell-antibody mixture was incubated at room temperature in the dark for 45 minutes. After washing in X-Vivo 15 medium, the cells were acquired on the BD FACSCalibur™ flow cytometer (BD Biosciences), and analysis was performed using BD FACSDiva™ software or FlowJo™ (Tree Star) software.

### RNA probe design

The probes for HIV gag were designed within 670 bases of the p24 coding region based on the consensus sequence among all HIV subtype B sequences for the corresponding region reported to the Los Alamos National Laboratory HIV database by 2009: cctatagtgcagaacctccaggggcaaatggtacatcaggccatatcacctagaactttaaatgcatgggtaaaagtagtagaagagaaggctttcagcccagaagtaatacccatgttttcagcattatcagaaggagccaccccacaagatttaaacaccatgctaaacacagtggggggacatcaagcagccatgcaaatgttaaaagagaccatcaatgaggaagctgcagaatgggatagattgcatccagtgcatgcagggcctattgcaccaggccagatgagagaaccaaggggaagtgacatagcaggaactactagtacccttcaggaacaaataggatggatgacaaataatccacctatcccagtaggagaaatctataaaagatggataatcctgggattaaataaaatagtaaggatgtatagccctaccagcattctggacataaaacaaggaccaaaggaaccctttagagactatgtagaccggttctataaaactctaagagccgagcaagcttcacaggaggtaaaaaattggatgacagaaaccttgttggtccaaaatgcgaacccagattgtaagactattttaaaagcattgggaccagcagctacactagaagaaatgatgacagcatgtcagggagtgggaggacc. The probes for the bcr and abl genes were each designed within 1,172 bases of the exon 1 sequence in the bcr gene (accession number NM_004327.3, probed region: nucleotides 779–1850) and 895 bases of sequences spanning multiple exons in the abl gene (accession number NM_005157.4, probed region: nucleotides 88–982). A schematic diagram of the probe region of each gene is shown in Figure S1. Target probes were custom designed for each target gene by Advanced Cell Diagnostics, Inc. (ACD) based on the algorithm described previously[27]. For fluorescence detection, the label probe for the target genes was conjugated to Alexa Fluor® 546 or 647. 18 s rRNA was selected as an internal RNA staining control, and the label probe was conjugated with FITC. All of the reagents, including the target probes and subsequent amplification probes, were manufactured by ACD as a custom order.

### *In situ* RNA analysis

For the slide based *in situ* RNA analysis, the previously published method was followed [27]. Briefly, cells were fixed on slides and digested with protease, followed by a series of hybridizations with the target-specific probes, amplifiers, and the fluorophore conjugated label probes. The slides were washed thoroughly with Wash Buffer (ACD, proprietary) after each hybridization step. Cells were then counterstained with DAPI (Life Technologies), treated with ProLong™ Gold anti-fade reagent (Life Technologies), and the images were imaged with a 40X or 60X objective on either the BD Pathway™ 435 bioimaging system (BD Biosciences) or an Olympus IX51 microscope containing filter sets from Semrock and Chroma Technology Corp®. Images were captured with a Hamamatsu digital CCD camera. BD AttoVision™ or Cell Profiler (www.cellprofiler.org) software was used for segmentation and image analysis (Fig. S2) [28], [29].

**Figure 1:**
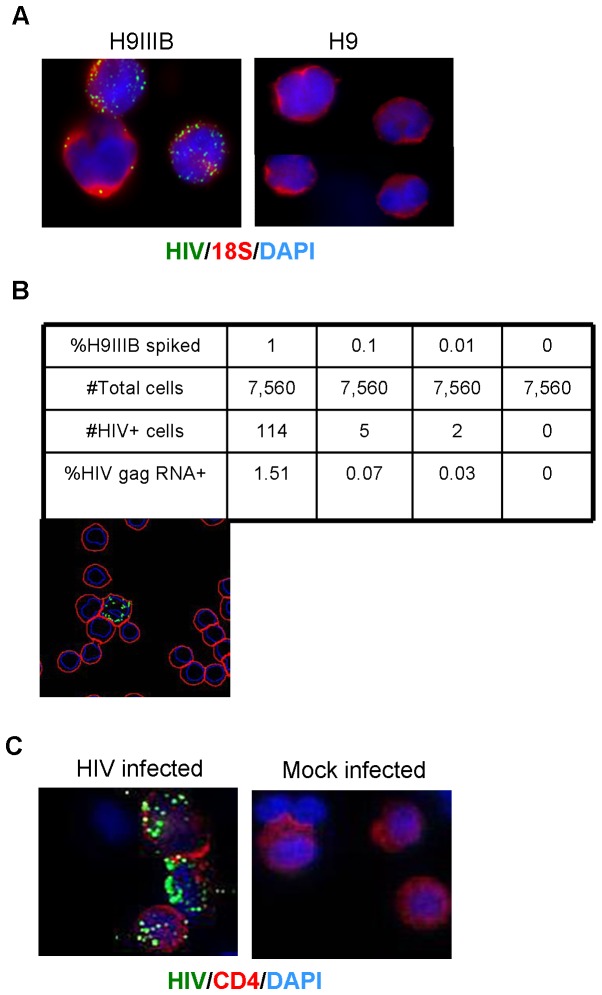
Detection of HIV gag RNA *in situ* in cell lines and PBMCs. (**a**) Representative color merged 60x images of the HIV+ cell line H9IIIB (left) and HIV negative cell line H9 (right); HIV gag RNA-Alexa Fluor® 647 (green), 18 s rRNA-FITC (red), and Nuclei-DAPI (blue). (**b**) The frequency of HIV gag RNA-positive cells within the 18 s+DAPI+ population was calculated after spiking HIV+ cells into the negative cell population at the stated percentages in the table. The image below the table is representative of data from image segmentation analysis. (**c**) 40x image of CD4 immunophenotyping overlaid with HIV gag RNA in HIV-infected PBMCs; Anti-CD4 antibody-Alexa Fluor® 488 (red), HIV gag RNA-Alexa Fluor® 546 (green), and DAPI (blue).

When immunophenotyping was combined with RNA detection (Fig. 1C), HIV-infected PBMCs were washed with PBS, and incubated with an anti-CD4 mAb conjugated to Alexa Fluor® 488 (BD Biosciences) in PBS. After a 30-minute incubation at room temperature, the cells were washed with PBS and imaged before proceeding with the *in situ* RNA staining procedure beginning with deposition and fixation on slides. Images were taken again after the RNA detection procedure. The two images were overlaid using the DAPI nuclear staining as a guide.

### RNA flow cytometry

The same procedure was applied for RNA flow cytometry as was used for the slide based *in situ* RNA detection as described previously, except that the cells were kept in microcentrifuge tubes throughout the staining process in a 100-µl volume for each hybridization step. The cells were centrifuged at 500 *g,* and washes were done in 1 ml of Assay Wash Buffer (ACD, proprietary). Acquisition was completed on either the BD FACSAria™ III (laser 488nm (filter 530/30 with 505 LP filter) for the detection of FITC, laser 561nm (filter 585/15) for the detection of Alexa Fluor®546 and laser 633nm (filter 660/20) for the detection of Alexa Fluor® 647), the BD FACSVerse™ (laser 488nm (filter 527/32 with 507 LP filter) for the detection of FITC and laser 640nm (filter 660/10) for the detection of Alexa Fluor® 647) , the BD FACSCanto II (laser 488nm (filter 530/30 with 502 LP filter) for the detection of FITC and laser 633nm (filter 660/20) for the detection of Alexa Fluor® 647), or the BD LSRFortessa™ (laser 488nm (filter 520/50 with 505 LP filter) for the detection of FITC and Alexa Fluor 488, laser 532nm (filter 582/15) for the detection of Alexa Fluor®546, and laser 640nm (filter 670/30) for the detection of Alexa Fluor® 647) (BD Biosciences). The BD FACSAria III was used for cell sorting. Sorted cells were deposited onto slides for image analysis, as described previously. All of the flow cytometry data analysis was performed using BD FACSDiva (BD Biosciences) or FlowJo (Tree Star) software.

### Statistical Analysis

Graphs and statistical analyses were completed using Microsoft® Excel® software.

## Results

### Specificity of *in situ* RNA detection

To evaluate the specificity of the RNAScope probes for *in situ* RNA detection, we designed an HIV gag-specific probe (Fig. S1). To visualize HIV gag-specific RNA detection, imaging cytometry was assessed. Slides were prepared with the H9 cell line chronically infected with the HIV-IIIB strain (H9IIIB) and with the HIV-uninfected cell line H9. The slides were hybridized with the HIV gag probe, followed by image acquisition and analysis. Figure S2 provides the details of the general segmentation and image analysis used for the experiments discussed in this article.

HIV gag RNA was detected as distinct fluorescent spots only in the HIV-infected H9IIIB cells, in contrast to the H9 cells, for which no detection of HIV gag RNA was observed (Fig. 1A). Using 18s ribosomal RNA (rRNA) as an internal control (depicted as red in the images in Fig. 1A), we identified and included only live cells and excluded dead cells and debris during image analysis (Fig. S2). When the HIV-infected cells were spiked into uninfected cells at various low frequencies (0.01 to 1%) prior to hybridization, a distinct gag RNA staining pattern was visible, as shown in the representative outline image after segmentation (Fig. 1B). A quantification of the HIV RNA+ cells, within the identified 18 s rRNA+DAPI+ cells, yielded the expected frequencies (table in Fig. 1B). HIV RNA signal was not detected in the control slides, where no target probes were used during the staining procedure. This data is representative of six independent experiments.

After demonstrating HIV RNA specificity with precision in cell lines, we next tested primary cells. Peripheral blood mononuclear cells (PBMCs) from normal uninfected individuals were stimulated and expanded for 3 days and then infected with HIV_NL43_ as described in methods. Briefly, PBMC blasts were infected and cultured until p24+ cells were readily detectable (within 3 to 10 days) by intracellular protein flow cytometry for HIVp24. HIV gag RNAs were seen only in the HIV-infected PBMCs and not in the mock-infected PBMCs (Fig. 1C). To confirm the CD4 specificity of HIV infection, cells also were stained with Alexa Fluor® 488 anti-CD4 antibody before the RNA staining procedure. HIV RNA was detected only in CD4-positive subsets of HIV-infected PBMCs, as expected [30], confirming the biological receptor specificity of HIV infection in this T-cell subpopulation (Fig. 1C).

### Comparison of RNA staining in cells on slides versus in suspension

**Figure 2:**
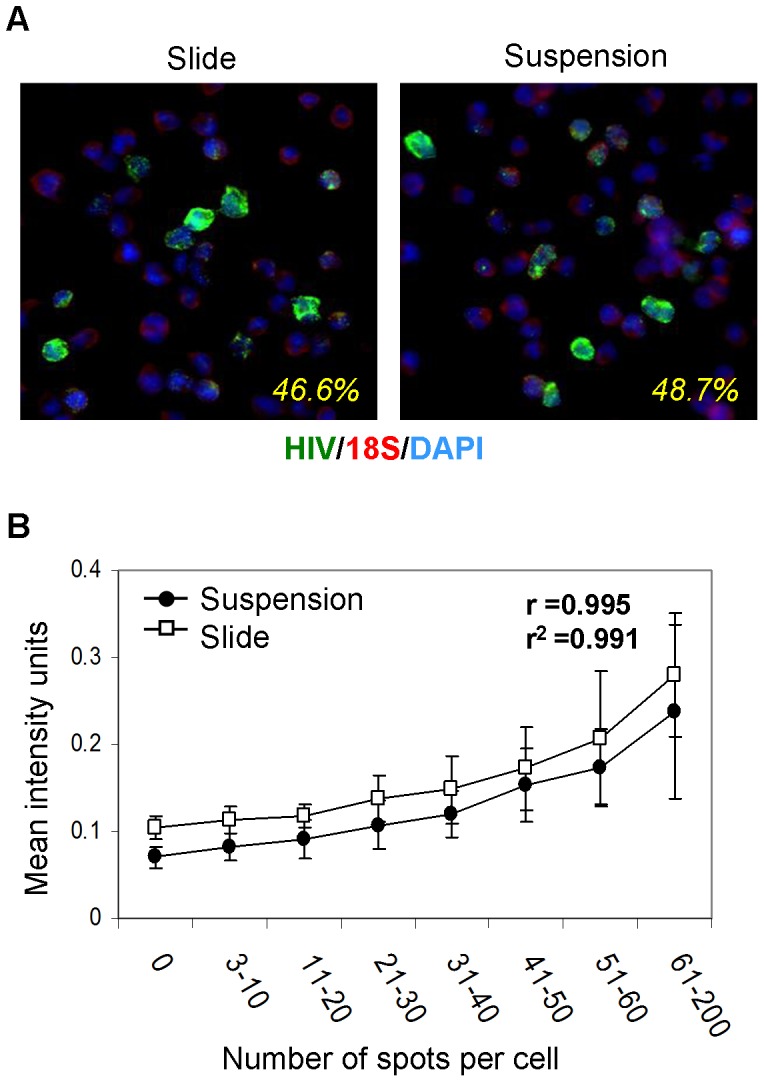
Comparison of HIV gag RNA *in situ* detection by a slide-based method vs a suspension-based method. (**a**) Pseudocolor-merged 40x images of a 1:1 mixture of H9 and H9IIIB cells via the slide-based RNA detection method (left) and the suspension method (right) of HIV gag RNA-Alexa Fluor® 546, 18 s rRNA-FITC, and DAPI. The calculated frequencies for HIV gag+18 s+ RNA for each are depicted in each image. (**b**) Mean intensity comparison of HIV gag for different HIV gag spot count ranges (bins) with the suspension-based and slide-based methods. Error bars depict the standard deviation within each spot count bin. The correlation coefficient, r, and the coefficient of determination, r^2^, are shown on the graph.

Once we established the specificity of the RNA staining in cells fixed on slides, we next wanted to determine whether the same RNA staining technology could be applied to cells kept in a suspension. A 1:1 mixture of H9IIIB and H9 cells was prepared and a portion of the cells was deposited and processed on slides. The remainder of the cells was kept in a suspension for the RNA staining procedure. The cells kept in suspension were processed as stated in methods. Briefly, the cells were processed in microcentrifuge tubes for the procedure, and the washes were performed by centrifugation and supernatant aspiration. After the RNA staining procedure, the cells in suspension were deposited onto slides for imaging. Both sets of slides were imaged and analyzed for HIV gag-positive cell frequency and HIV gag signal intensity within nucleated live cells. The punctuate staining pattern in both methods was visually similar, and the percentage of HIV+ cells in both cases was very near the expected 50% (Fig. 2A). The mean fluorescence intensity (MFI) of the HIV RNA signal in the suspension stained cells was slightly lower than that of the cells stained on the slides, though still comparable. Not surprisingly, for both preparation methods, the distribution of the number of spots per cell was highly correlated with the distribution of cellular fluorescence intensity, as evident from the correlation coefficient being close to 1 (Fig. 2B). This data was reproduced and also confirmed using bcr Alexa Fluor® 647 and abl Alexa Fluor® 546 in the K562 cell line.

### Analysis of intracellular RNAs by flow cytometry

**Figure 3:**
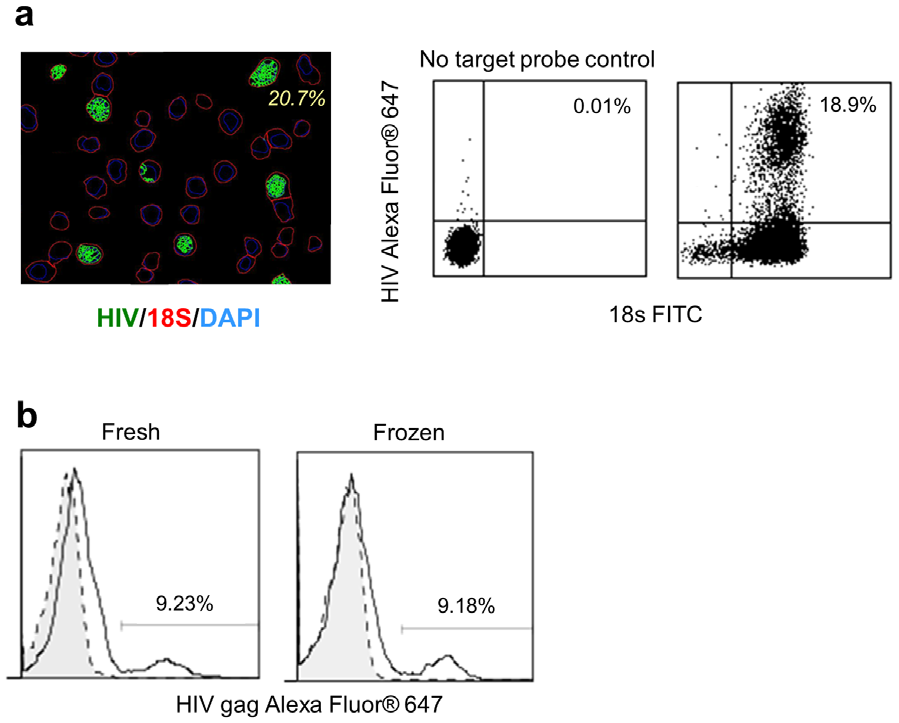
Validation of the RNA flow cytometry procedure. (**a**) A pseudocolor merged segmentation mask image obtained using Cell Profiler software analysis of a mixture of H9 and H9IIIB cells (left image); HIV gag-Alexa Fluor® 647, 18 s-FITC, and DAPI counterstain. The calculated frequency of HIV gag+ cells (within the 18 s+DAPI+ cells) is shown on the image. The RNA flow cytometry plots of the same mixture of H9 and H9IIIB cells. (**b**) Flow cytometry overlay histograms of HIV gag RNA in HIV-infected PBMCs (solid line) and mock-infected PBMCs (tinted with dashed line) in freshly acquired PBMCs (left plot) and the same PBMCs after cryopreservation (right plot).

Having obtained similar RNA staining results with both the slide and the suspension- based assays using imaging, we next set out to establish whether the stained suspension cells could be acquired on a flow cytometer with a specificity and sensitivity similar to that with imaging. A mixture of H9IIIB and H9 cells was prepared, which contained approximately 20% HIV+ cells, to compare the results from two platforms, imaging and flow cytometry. The mixed cell suspension was stained for HIV gag RNA and 18 s rRNA and then divided into two portions. One portion of the stained cells was deposited onto a slide, and images were acquired. The remaining cells were acquired in suspension on the flow cytometer. Quantitative analysis of the two preparations yielded the anticipated result: 20.7% and 18.9% of the cells were defined as HIV+ by image and flow analysis, respectively (Fig. 3A). A control that omitted target probes during the RNA staining procedure was used to assess nonspecific staining (data not shown) and to set the negative gates for the flow cytometry analysis (Fig. 3A). Similar results were observed when the HIV gag Alexa Fluor® 546 label probe was used in place of Alexa Fluor® 647 in a separate experiment (data not shown).

To further investigate RNA flow cytometry in primary cells, *in vitro* HIV-infected PBMCs were prepared as described in methods. A mock-infected sample was included as a control. Since it is often important to be able to ship primary samples, and to be able to analyze from previously frozen samples for retrospective studies, we also analyzed the effects of cryopreservation on these HIV-infected and mock-infected PBMCs. A significant HIV gag RNA-positive population was seen in the RNA analysis of both fresh and cryopreserved infected samples (solid lines) compared to mock-infected controls (dashed lines) (Fig. 3B, data is representative of eight independent experiments).

### Cellular mRNA analysis and multiplex RNA flow cytometry

To evaluate the feasibility of RNA flow cytometry beyond the HIV target, we designed RNA target probes for messenger RNAs of the bcr and abl genes (Fig. S1). The performance of these target probes was evaluated in the K562 cell line expressing the bcr-abl fusion construct [31]. The bcr and abl targets were tested individually as well as together. Each target was clearly detectable by flow cytometry analysis when individually tested as well as when the target probes were combined (Fig. S3A).

**Figure 4:**
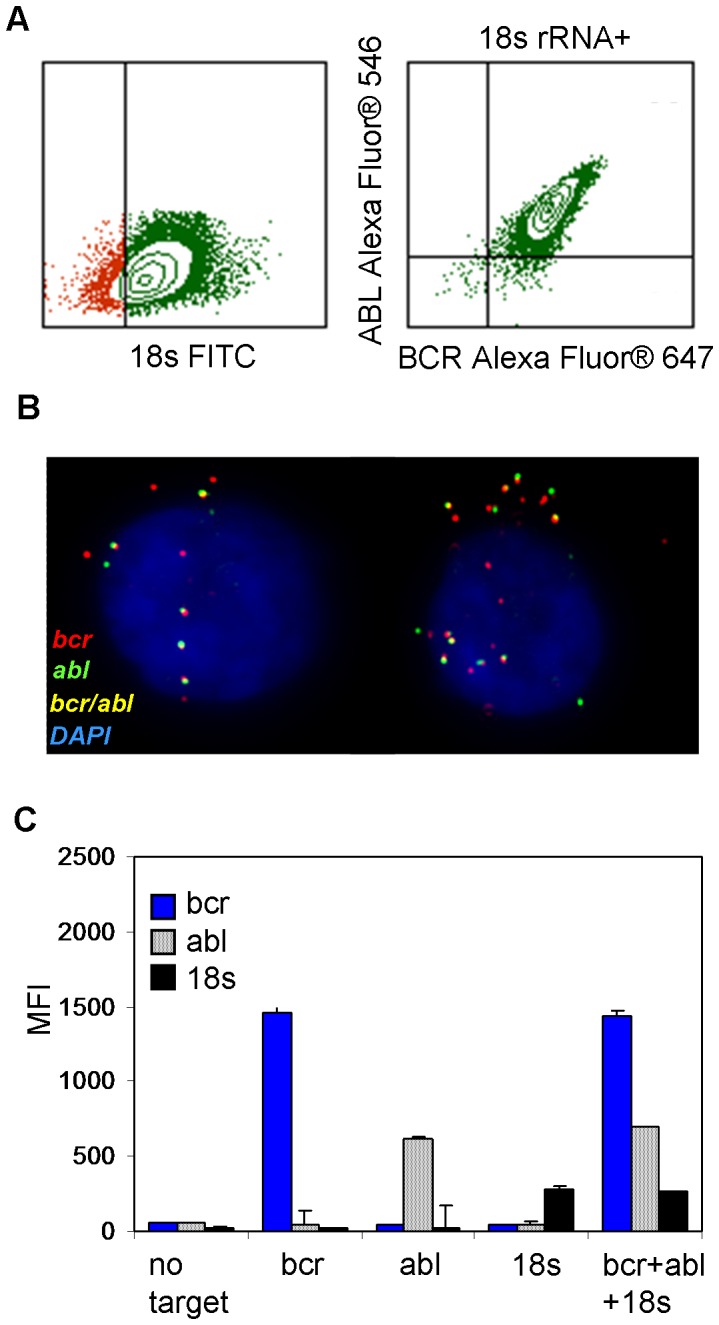
Assessment of RNA flow cytometry probe multiplexing. (**a**) Flow cytometry plots from three-probe (bcr, abl, 18 s) RNA analysis in the K562 cell line: 18 s rRNA (left) and bcr vs. abl RNA in the 18 s+ events (right). (**b**) 60x pseudocolored images of sorted bcr+abl+18 s+ cells from K562 cells; Alexa Fluor® 647 labeled bcr (red), Alexa Fluor® 546 labeled abl (green). The bcr/abl fusion transcripts are shown in yellow due to the merging of both the Alexa Fluor® 546 and Alexa Fluor® 647 dyes. Cells were counterstained with DAPI. (**c**) Graph showing the effects of RNA staining by multiple probes (bcr+abl+18 s) compared to a single target probe on MFIs in RNA flow cytometry. Bars represent the standard deviation for duplicate samples run.

Utilizing the bcr, abl, and 18 s RNA target probes, we then assessed the multiplexing capability of RNA flow cytometry in the K562 cell line. In the multiplex analysis, a clear population of bcr and abl double-positive cells was identified within the 18 s rRNA+ gated cell population (Fig. 4A). One no-target probe control was used to set the negative gates for all of the targets. When the bcr+abl+18 s+ cells were subsequently sorted and imaged on a slide, closely paired spots likely representing the mRNA of bcr-abl fusion constructs were clearly visible within the cells (yellow in Fig. 4B). The ratio of yellow bcr-abl fusion spots to green abl spots confers with our qRT-PCR data for the ratio of fusion transcripts to abl mRNA quantitation (data not shown). These data suggest that the yellow spots visualized in these slides post sort are indeed bcr-abl fusion RNA, which validates the potential for subcellular analyses of gene expression using the cell sorting function of flow cytometry.

MFI data generated by flow cytometry analysis of each individual RNA target was compared with a multiplex sample containing all three targets (bcr, abl, and 18 s). We observed no apparent difference in the MFI when a single target probe was added or when all three probes were added (Fig. 4C). Additionally, in the multiplex analysis of bcr, HIV gag, and 18 s RNAs in the HIV-negative K562 cell line, there was no indication of interference when a non-relevant probe (HIV gag) was included with the specific target probes (18 s and bcr) during the hybridization process (Fig. S3B). Overall, there was no indication of interference among the different probe sets during the signal amplification process in the multiplex RNA flow cytometry analysis.

### Sensitivity of RNA flow cytometry

**Figure 5:**
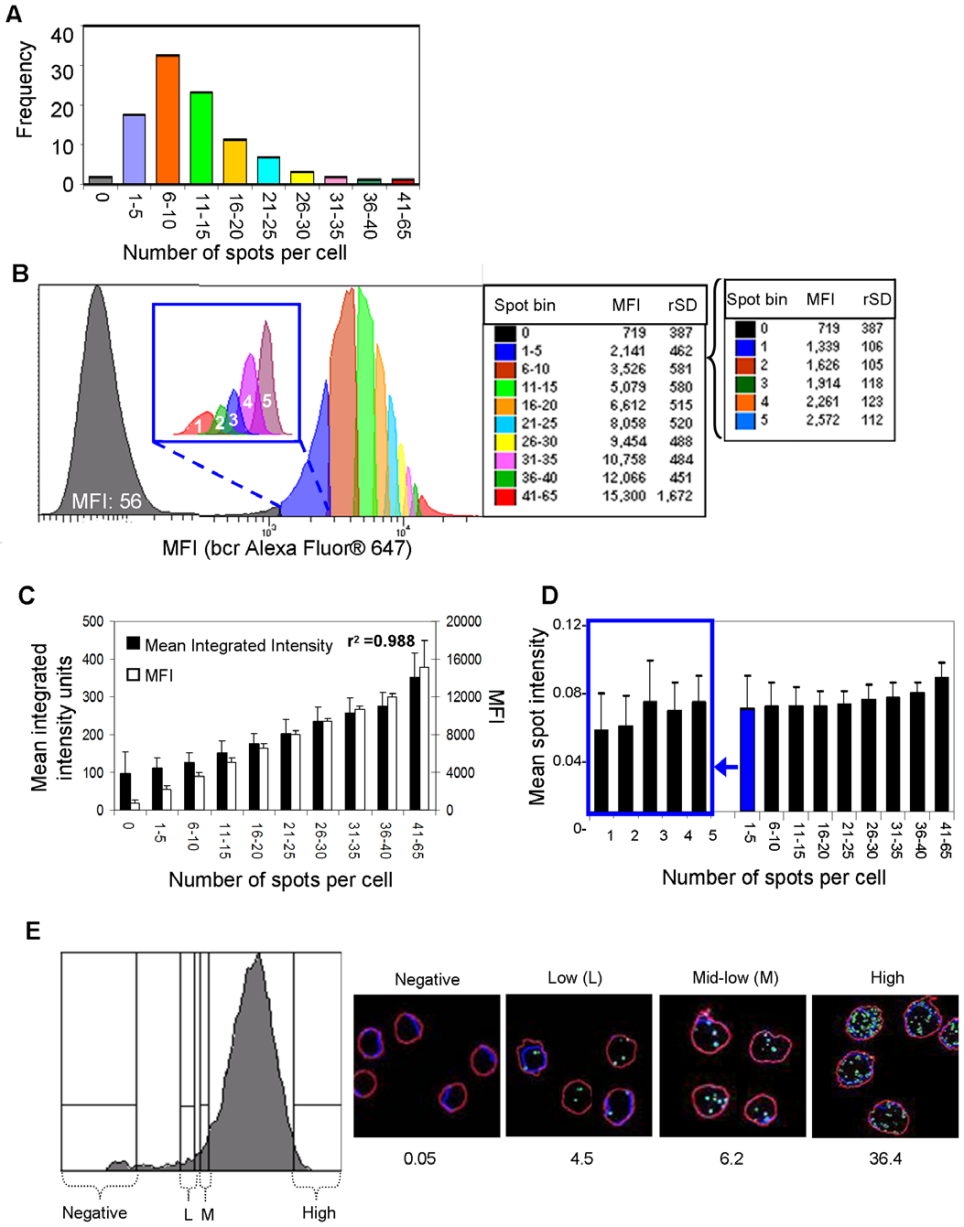
Validation of the sensitivity of RNA flow cytometry. (**a**) Breakdown of spot numbers from image analysis (bcr+18 s+DAPI+) into bins (x-axis) with corresponding frequencies (y-axis). (**b**) RNA flow histogram of K562 cells split into groups using the percentages obtained in Figure 5A with the corresponding MFIs (x-axis and table). Colors correspond to the spot bin numbers in the bar chart in 5a. The lowest bin (spot count 1–5) was further subdivided and is shown in the inset on the histogram with the corresponding spot count on each peak. (**c**) Comparison chart of mean integrated intensities (black bars) obtained from image analysis and MFIs (white bars) obtained from RNA flow cytometry analysis (5b). Standard deviations are shown for each group. The coefficient of determination (r^2^) is displayed on the graph (0.988). (**d**) Spot intensities for each spot bin with correlating standard deviations for each group. The lower bin (1–5) was further subdivided and is shown on the left side of the chart. (**e**) Histogram showing sort gates for the different bcr MFI subsets in the K562 cell line. Outline overlay images shown to the right of the histogram are representative of the sorted cells from each subset sorted showing bcr RNA (green), 18 s rRNA (red), and DAPI counterstaining (blue). The bcr spot count average per cell is listed below its respective image.

To further evaluate RNA flow cytometry for the analysis of low-abundant intracellular RNA, the detection sensitivity was assessed using bcr as the target RNA in the K562 cell line. Given that RNA staining was comparable between cells on slides and cells in suspension (Fig. 2), we directly compared the MFI data obtained by flow cytometry with the RNA spot counts calculated by image analysis from the same original cell suspension. K562 cells were stained in suspension with target probes for bcr, a portion of the cells was deposited on a slide, and images were acquired. The remaining cell suspension was acquired on the flow cytometer. RNA spots for the bcr target were calculated from image analysis, and spot frequencies were grouped into bins, to segment the data points for analysis (Fig. 5A). The respective percentage of cells within each bin was then applied to the corresponding flow cytometry data. This was done by setting the analysis gates in the flow histogram on the bcr RNA-positive cells (Fig. 5B) such that each gate’s frequency was equivalent to the percentage calculated from the image spot count analysis (y-axis values from Fig. 5A). The corresponding MFI for each gate could then be obtained in the flow cytometry analysis (table in Fig. 5B). To assess the detection sensitivity of RNA flow cytometry, the lowest spot count bin (1–5 range) was further split into individual spot counts and the MFIs were determined (inset to the table in Fig. 5B). From this analysis it was determined that the cells containing one or two RNA spots could be distinguished using RNA flow cytometry. Considering that each RNA is detected as an individual spot by the RNAScope slide based assay and that this assay was shown to be a single molecule detection method with a signal detection efficiency of 85% [27], this data indicates that cells containing very few RNA copies are able to be distinguished from the background.

When the MFIs from the flow cytometry analysis (Fig. 5B) were plotted against the mean integrated intensities from the imaging analysis for each bin, we found a very good correlation between the two measurements (r^2^ value of 0.988, Fig. 5C). Additionally, the individual spot intensities were found to be very consistent among the different spot count bins, even at the lower end of the detection limit (Fig. 5D). When we sorted different subsets of bcr RNA-positive cells, based on MFI, and subsequently deposited the sorted cells onto slides for RNA image analysis (Fig. 5E), the spot frequency correlated with the MFI from the flow histogram (Fig. 5E). This provides confirmation that the MFIs obtained in RNA flow cytometry acquisition correspond to the HIV spot numbers and intensities obtained in RNA slide image analysis.

## Discussion

Over the last few decades, flow cytometric detection of intracellular RNAs has been attempted by applying various molecular technologies [15]–[17], [32]. However, the specificity and/or sensitivity of all previous attempts have not been suitable for the wide range of intracellular RNA analyses, particularly in the case of low abundance of target RNA sequences. Here we have described a method for RNA flow cytometry with performance characteristics enabling the simultaneous analysis of multiple RNAs in individual cells at the sensitivity-detection limit for a single RNA molecule. By adapting a novel signal amplification technology [27] that provides superb background suppression with its unique target specific probe design, we could analyze the highly amplified signal of even low frequency specific mRNAs by flow cytometry without increasing the background signal.

We have established the quantitative capabilities of RNA flow cytometry by demonstrating that the signal amplification per each RNA molecule is quantitatively consistent among all cells in a population (Fig. 5D). Furthermore, the imaging cytometry data from RNA amplification on slides correlated with the flow cytometry analysis on suspension cells. The amplified signals were highly specific for each target RNA, as evidenced in the HIV gag model, with little or no signal from the HIV-uninfected cells or cells treated with either no or irrelevant target probes (Fig 3A; Fig. S3B). Furthermore, we demonstrated the sensitivity of RNA flow cytometry to be applicable for the analysis cells containing very low RNA copies in each cell (Fig. 5B). In this study, up to three targets in multiplex RNA flow cytometry have been validated (Fig. 4A). However, we believe that the level of multiplexing could be further expanded for a higher complexity gene expression analysis, since no sign of inter-target interference during the signal amplification was observed, even when the two target RNAs were in close proximity, as we demonstrated with the bcr-abl fusion transcript (Fig. 4B).

To the best of our knowledge, this is the first demonstration of a sensitive flow cytometric analysis of specific messenger RNA in individual cells. The high sensitivity and specificity of the presented method enable the analysis of RNA molecules present in low copy numbers from individual cells and in minority subpopulations. Additionally, RNA flow cytometry will be extremely valuable in rare cell analyses that involve both dynamic and heterogeneous populations of cells. This single cell RNA analysis method can be applied to various types of cellular samples, including cell lines, primary cells, and even previously cryopreserved cells (Fig. 3B).

We believe that RNA flow cytometry, as demonstrated in this study, represents a novel tool to validate gene expression profiles in individual cells or for a comprehensive evaluation of the expression dynamics of genes from different cell types. It will be particularly valuable for understanding the complex network of pathways in cells among very heterogeneous cell populations and might lead to widespread applications in areas including stem cell biology, oncology, immunology and immune cell-related diseases, and developmental biology. A combined RNA flow cytometry analysis with protein targets, such as the preliminary data shown in Figure 1B, would be a highly desirable future application, in cases when the subsets of cells have already well defined antigen expression.

## Supporting Information

Figure S1 **RNA target-specific probe design.** (**a**) Schematic diagram for the HIV RNA probe location and (**b**) for bcr and abl probe locations (based on the p210 fusion transcript).
(TIF)

Figure S2 **Image analysis example.** Original images were analyzed using Cell Profiler software. (**a**) Raw image data from DAPI, 18 s, and bcr stained K562 cells. (**b**) Using Cell Profiler software for analysis, after background subtraction, segmentation was done on cells using DAPI nuclear staining (left image, green outline) and then the cells were further segmented based on 18 s staining (second image, red outlines). Only those cells with both DAPI and 18 s staining were included in the spot count. Bcr spots (third image, green) were first enhanced in the software and then related to a particular cell and counted. The right image is representative of the merged pseudocolored image resulting from the analysis. (**c**) Example of the resulting data from the analysis in b. For quantitative analysis, additional manual evaluation and adjustment have been applied when multiple cells were deposited closely and obscured the segmentation boundary.
(TIF)

Figure S3 **RNA flow cytometry control experiment plots.** (**a**) bcr Alexa Fluor® 647 (x-axis) vs abl Alexa Fluor® 546 (y-axis) in K562 cells with only the bcr target probe included (left), only the abl target probe included (middle,) and both bcr and abl probes included (right). (**b**) HIV gag Alexa Fluor® 546, a non-relevant target, and bcr Alexa Fluor® 647 in K562 cells where no target probes were included (left plot) and where both probes in addition to 18 s rRNA FITC, were included, showing the lack of non-specificity of the probes when the target is absent.
(TIF)
