# Supplementary figures and images for: Detection of Low Abundance RNA Molecules in Individual Cells by Flow Cytometry
Source: PLoS One. 2013 Feb 18;8(2):e57002. doi: 10.1371/journal.pone.0057002 (PMC3575505; doi:10.1371/journal.pone.0057002)

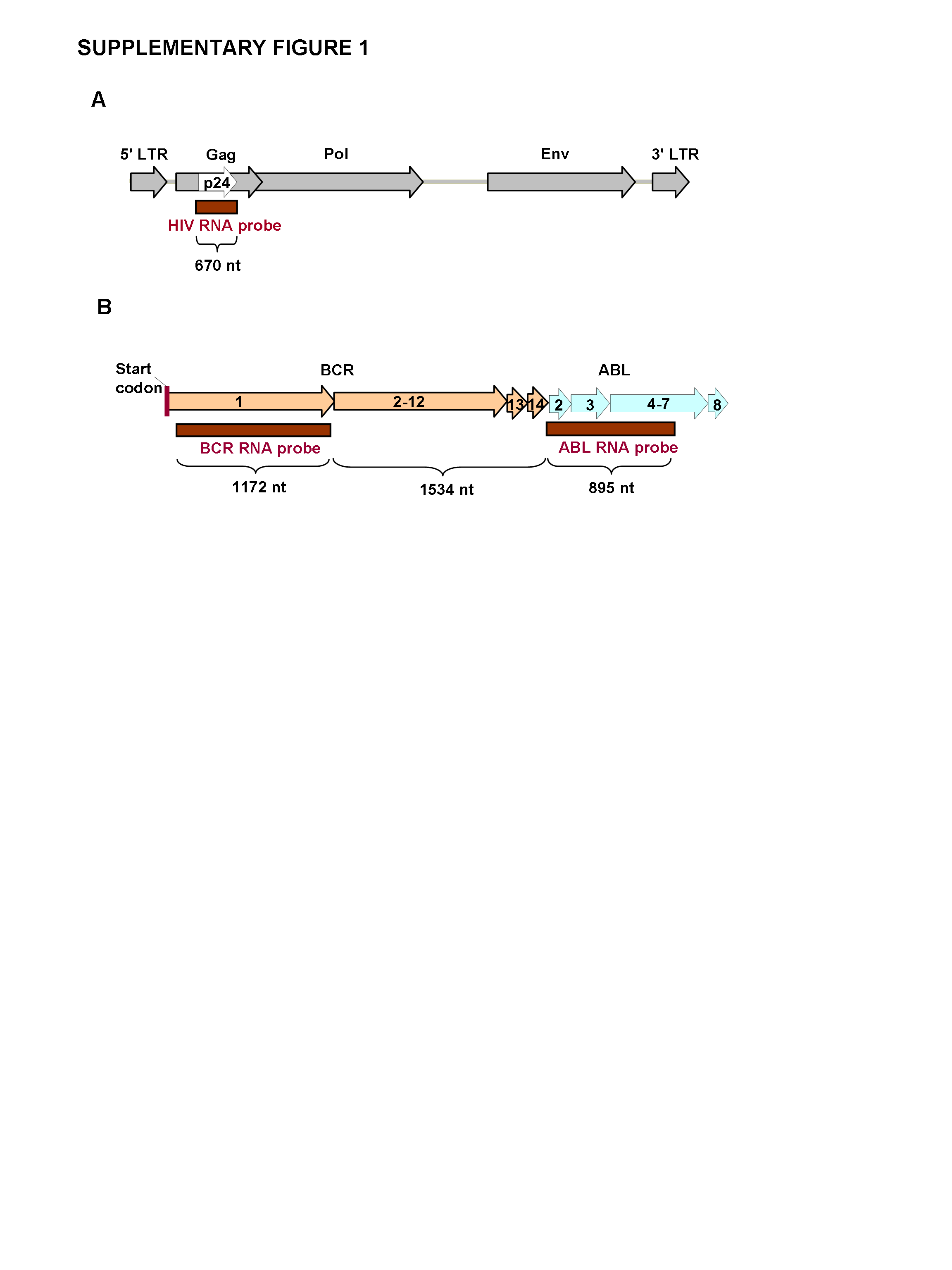

Supplement: Figure S1 — RNA target-specific probe design. (a) Schematic diagram for the HIV RNA probe location and (b) for bcr and abl probe locations (based on the p210 fusion transcript). (TIF) [file pone.0057002.s001.tif]

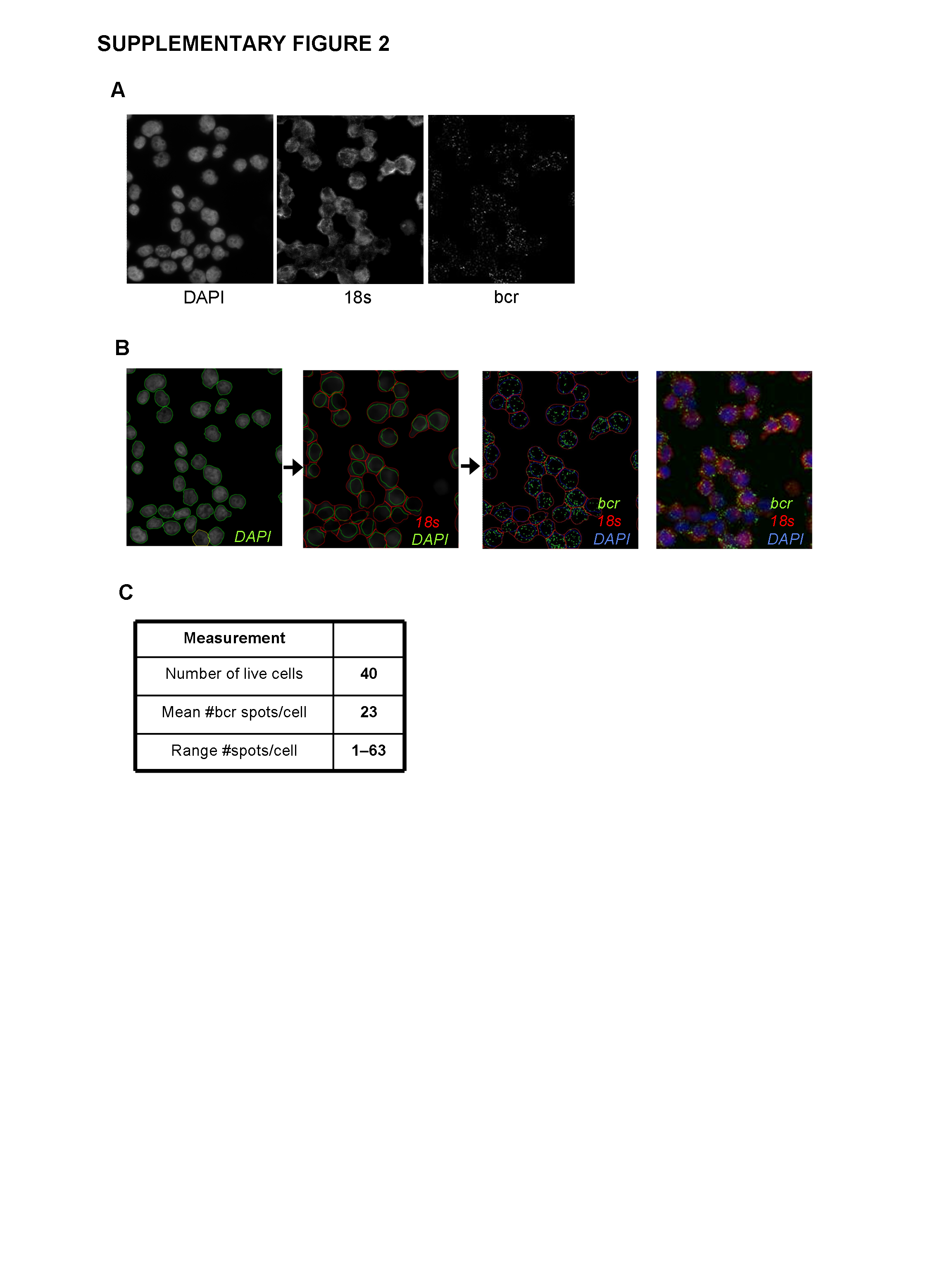

Supplement: Figure S2 — Image analysis example. Original images were analyzed using Cell Profiler software. (a) Raw image data from DAPI, 18 s, and bcr stained K562 cells. (b) Using Cell Profiler software for analysis, after background subtraction, segmentation was done on cells using DAPI nuclear staining (left image, green outline) and then the cells were further segmented based on 18 s staining (second image, red outlines). Only those cells with both DAPI and 18 s staining were included in the spot count. Bcr spots (third image, green) were first enhanced in the software and then related to a particular cell and counted. The right image is representative of the merged pseudocolored image resulting from the analysis. (c) Example of the resulting data from the analysis in b. For quantitative analysis, additional manual evaluation and adjustment have been applied when multiple cells were deposited closely and obscured the segmentation boundary. (TIF) [file pone.0057002.s002.tif]

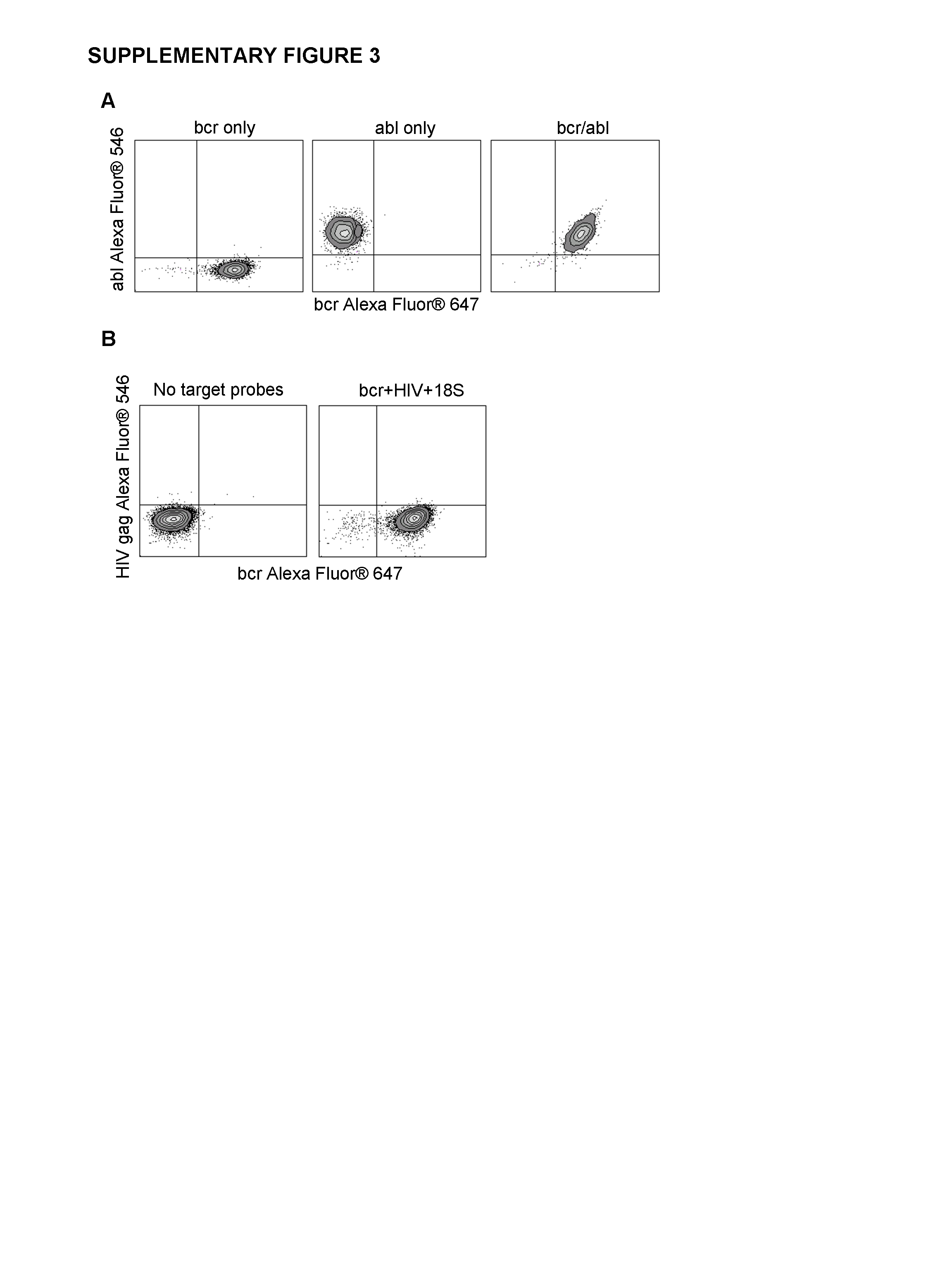

Supplement: Figure S3 — RNA flow cytometry control experiment plots. (a) bcr Alexa Fluor® 647 (x-axis) vs abl Alexa Fluor® 546 (y-axis) in K562 cells with only the bcr target probe included (left), only the abl target probe included (middle,) and both bcr and abl probes included (right). (b) HIV gag Alexa Fluor® 546, a non-relevant target, and bcr Alexa Fluor® 647 in K562 cells where no target probes were included (left plot) and where both probes in addition to 18 s rRNA FITC, were included, showing the lack of non-specificity of the probes when the target is absent. (TIF) [file pone.0057002.s003.tif]
